# Multiply Perturbed Response: A Computational Protocol to Identify Cooperative Allosteric Residue Combinations Driving Protein Conformational Transitions

**DOI:** 10.21769/BioProtoc.5718

**Published:** 2026-07-05

**Authors:** Kübranur Kazan, Melike Berksoz, Burak Kocuk, Ali Rana Atilgan, Canan Atilgan

**Affiliations:** Faculty of Engineering and Natural Sciences, Sabanci University, Istanbul, Turkey;

**Keywords:** Multiply perturbed response, Conformational transition, Allosteric residue combinations, Elastic network model, Linear response theory, Overlap maximization, Variance-covariance matrix

## Abstract

Protein function often depends on dynamic conformational transitions driven by external factors or molecular interactions. Understanding the allosteric mechanisms underlying these transitions is essential for mechanistic insight into protein function. Molecular dynamics (MD) simulations are widely used to study protein dynamics; however, capturing large-scale, rare transitions is computationally expensive. To address this, we previously developed Perturbation Response Scanning (PRS), based on elastic network models and linear response theory, but PRS is limited in capturing collective effects because it perturbs one residue at a time. Here, we present Multiply Perturbed Response (MPR), which extends PRS by applying simultaneous perturbations to multiple residues to identify allosteric residue combinations that drive conformational transitions. This protocol provides a workflow for structure preparation, displacement, and covariance-matrix calculations, overlap analysis, and visualization. It can be applied to static structures or trajectories from MD simulations, requiring initial and final protein structures as the main inputs and an optional MD trajectory for trajectory-based analysis. The main outputs are residue combinations that maximize overlap, *O*_max_ values, corresponding force vectors, and visualization files. These outputs help identify cooperative allosteric regions and residues for mechanistic interpretation or further experimental validation. By perturbing multiple residues simultaneously, MPR captures conformational transitions arising from combined residue effects. The method is easy to use, reproducible, and accessible through open-source tools and libraries.

Key features

• Facilitates the identification of multiple allosteric hotspot residues using optimized multi-residue perturbations.

• Extends classical perturbation-response scanning to account for the coordinated effects of multiple simultaneous perturbations.

• Enables structure-based and trajectory-based analyses within a single framework, using static structural information or MD-derived covariance information.

• Provides interpretable outputs, including ranked residue combinations, overlap values, optimized force vectors, and ChimeraX-compatible visualization files.

## Graphical overview


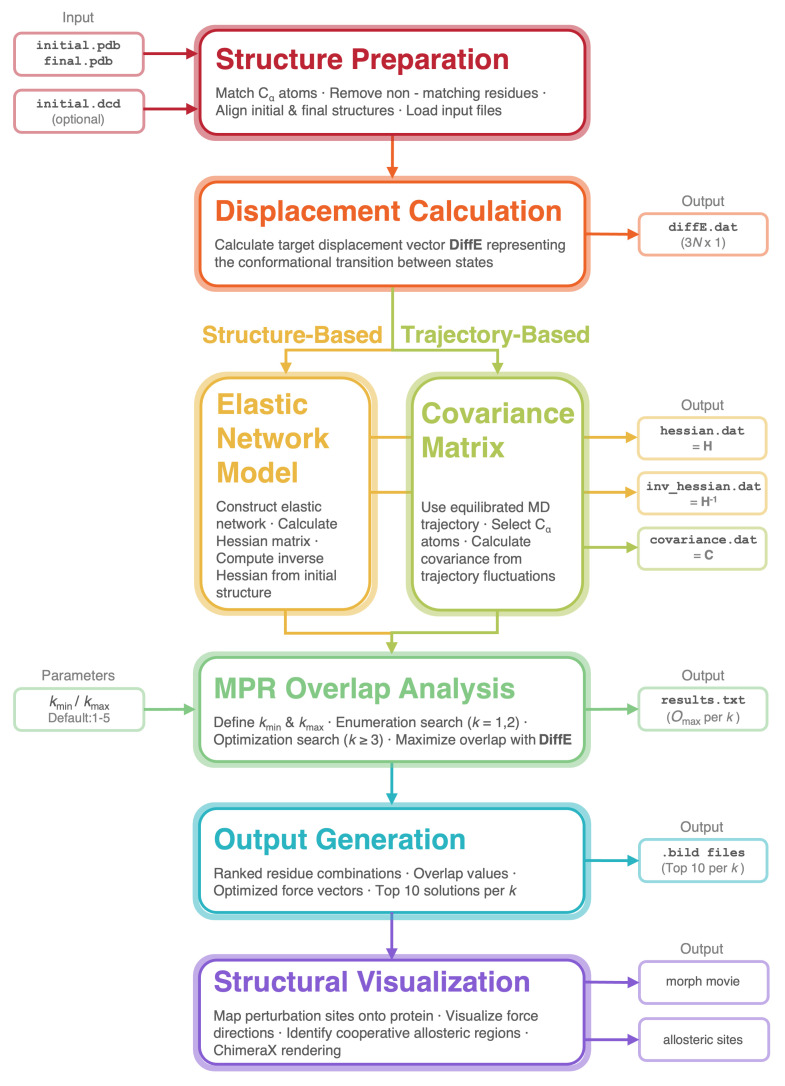


**The Multiply Perturbed Response (MPR) workflow identifies allosteric residue combinations that drive protein conformational transitions using structure- or trajectory-based response matrices**

## Background

Proteins are dynamic molecules that adopt multiple conformations. Protein structure is closely linked to how it responds to internal factors and interactions with the external environment, which in turn determines function in diverse biological processes. The energy landscape of a protein describes the relative free energies of its possible conformations; the probability of occupying a given conformation is reflected by the depth of that state on the landscape. Factors such as ligand binding [1], pH [2], ionic strength [3], or mutations [4] can reshape the landscape and drive structural changes [5]. The physicochemical properties of amino acids also influence these responses. Charged, polar, or hydrophobic residues can affect local interactions, packing, and collective motions [6,7]. In this context, our research focuses on identifying allosteric regions. Allostery refers to a perturbation in one region of a protein that leads to structural and dynamic changes in distant regions, translating a local signal into a global functional response.

Molecular dynamics (MD) simulations are widely used to study protein dynamics and allostery; however, observing large and rare conformational transitions often requires long simulation times and substantial computational resources. In some cases, directly observing the effects of environmental conditions is not feasible, which limits the applicability of purely MD-based approaches [8]. Alternative approaches have been developed to overcome these limitations. For example, elastic network models (ENMs) and methods based on linear response theory have been introduced. In the ENM framework, proteins are represented as coarse-grained networks (typically at the C_α_ level), enabling the simple yet efficient computation of residue-level fluctuations and their modal decompositions [9–11].

Perturbation Response Scanning (PRS) is a widely used computational method for identifying allosteric residues, grounded in linear response theory [12]. This framework assumes that structural responses near equilibrium are linearly related to applied forces [13]. Accordingly, in PRS, proteins are coarse-grained at the C_α_ level and subjected to systematic single-residue perturbations to identify residues whose perturbations best reproduce experimentally observed conformational changes [14]. PRS has been successfully applied to various proteins to identify functional hotspots, including enzymes (Caspase-6, ADK) [15,16], molecular motors (Kinesin, Myosin) [16], signaling proteins (CheY, PDZ) [17,18], viral proteins (HIV-1 RT) [16], and many others. Interestingly, these studies have revealed that the residues that maximize the overlap with the identified conformational change are not limited to surface-exposed positions; depending on the system, they may include exposed residues, partially buried interface residues, or buried residues near functional cores. However, PRS is practically limited to single-residue perturbations, and it may not detect conformational changes caused by the simultaneous and collective effects of multiple residues. When it comes to domain or hinge-movements, or rearrangements dependent on environmental conditions, multiple coordinated perturbations may be required to trigger the observed structural rearrangements [16].

To overcome this limitation, we introduce the Multiply Perturbed Response (MPR) method [19]. This approach extends PRS by applying forces simultaneously to multiple residues, capturing conformational transitions driven by collective residue effects. When only static initial and final structures are available, the protocol can be executed using the structure-based formulation alone. When MD trajectories are provided, the trajectory-based method captures equilibrium conformational fluctuations, improving the precision of the predicted conformational response. We note that for proteins without experimentally determined structures, MPR can be applied using predicted structural models. In this case, alternative conformational states may be generated using AlphaFold/ColabFold with different MSA depths, RoseTTAFold, ESMFold, or other validated structure-prediction workflows. However, because the input conformations are computational predictions, the resulting MPR residues should be interpreted as hypotheses for future experimental testing rather than experimentally validated allosteric sites.

MPR integrates enumeration- and optimization-based strategies to explore solutions across different numbers of perturbed residues (*k* ≥ 1). Enumeration enables exhaustive identification of optimal residue combinations for small *k* values, while optimization-based formulations allow efficient exploration of higher-order perturbations without prohibitive computational costs. As a result, the method substantially reduces computational expense compared with fully atomistic approaches while retaining sensitivity to collective allosteric effects. Using open-source software and widely used bioinformatics libraries increases the accessibility and applicability of the method.

## Software and datasets

1. Script/Code: MPR (Multiply Perturbed Response), v1.0.0, 2025, open source, free

2. Software: ProDy, v2.4.1, August 2025, MIT License, free

3. Software: Biopython, v1.86, October 2025, BSD 3-Clause License, free

4. Software: ChimeraX, v1.11, December 2025, GNU GPL 3.0 License, free for academic use

5. Software: AlphaFold2 (via ColabFold), ColabFold v1.5.5 and AlphaFold2 v2.3.2, April 2023, MIT License (ColabFold), Apache 2.0 License (AlphaFold2), and CC BY 4.0 License (associated model parameters and database contents), free

6. Software: AlphaFold3, v3.0.1, January 2025, CC-BY-NC-SA 4.0 License, free for academic use

7. Software: Gurobi Optimizer, v13.0.1, November 2025, Gurobi Academic License, free for academic use; restrictions apply. Gurobi Optimizer is used for optimization-based MPR calculations. Open-source alternatives such as HiGHS, SCIP, or GLPK may be possible, but they would require code adaptation and have not been systematically tested in the current implementation

8. Software: Python v3.8 is recommended

9. Data: RCSB Protein Data Bank, free access

The MPR code used in this protocol is freely available on GitHub at https://github.com/midstlab/MPR_Bio-Protocol. The repository includes a detailed README file with installation instructions, required input formats, example runs, expected output files, parameter descriptions, and explanations of the notebook used in the workflow. The repository also provides an environment.yml file to reproduce the recommended Python environment.

All analyses described in this protocol can be reproduced using this repository together with Biopython, ProDy, and ChimeraX. Optimization-based MPR analyses require Gurobi Optimizer or adaptation to an alternative solver. The required software is available through free or academic-use licenses.

**Software setup**

1. Download the MPR code from the MidstLab GitHub page.

2. Create the recommended Python environment using the environment.yml file provided in the GitHub repository. This file provides the Python 3.8 environment required for the ProDy-based structure calculations. Alternatively, install the required packages manually in a Python 3.8 environment.

3. Install the ProDy package, which is used for elastic network modeling and Hessian calculations based on linear response theory [20].

4. Install Biopython to perform structural alignment and overlapping operations [21].

5. Install ChimeraX for three-dimensional visualization of force vectors and protein conformational transitions [22].

6. Install Gurobi Optimizer if optimization-based MPR analysis is required; enumeration-based analyses for small *k* values can be performed without Gurobi [23]. Gurobi is not included in environment.yml because its Python version compatibility depends on the platform and installation method.

7. Place the MPR code in the working directory specified by the user.

## Procedure

This MPR protocol describes a computational workflow for identifying allosteric residues that collectively drive known protein conformational transitions. The procedure supports two complementary strategies for describing protein mechanics: (i) a structure-based approach that relies solely on static structural information and (ii) a trajectory-based approach that incorporates time-dependent information from molecular dynamics simulations. In both cases, the workflow includes structure preparation, definition of the displacement vector, construction of the relevant matrix (inverse Hessian or covariance), overlap maximization, residue selection, and structural visualization. All steps are designed to be reproducible using the provided notebook and open-source software.

**A. Structure preparation**

*Note: This section covers the preparation of structures for subsequent MPR calculations.*

1. Obtain the initial and final structures of the protein.

a. If experimental structures are available, retrieve them from the Protein Data Bank (PDB).

b. If experimental structures are unavailable in the PDB, predict the structures using AlphaFold2 (via ColabFold), AlphaFold3, or alternative structure prediction tools such as RoseTTAFold and ESMFold [24–28].

c. Using ColabFold with reduced MSA settings is preferable to obtain alternative, open (*apo*-like) conformations, as deep MSAs may bias predictions toward closed (*holo*-like) conformations [29,30].

2. Ensure that the initial and final structures contain the same number of C_α_ atoms and consistent residue ordering and indexing. If the structures contain mismatched residues, missing terminal residues, unresolved loops, or different chain lengths, retain only the common residue range. Trim additional terminal residues that are present in only one structure.

**Caution:** When examining the results, consider that the index starts at 1; the actual residue numbers of the allosteric residues should be calculated relative to the PDB's starting residue number.

**Caution:** Even a small mismatch at the termini can cause deviations in displacement vectors and affect overlap calculations.

3. Place the initial and final PDB files in the working directory.

**B. Displacement and covariance matrix calculation**

*Note: In this section, the displacement vector and the inverse Hessian (in the structure-based approach) or covariance matrix (in the trajectory-based approach) that form the basis of MPR are calculated.*

1. Continue the subsequent steps in the working directory containing the MPR code and files.

2. Activate the Python environment required by the pipeline.

3. All dependencies should be correctly installed.

4. Open the MPR.ipynb notebook and, if required, replace the initial structure, final structure, and trajectory file names.

5. The initial and final structures are superimposed using the Biopython SVDSuperimposer tool based on C_α_ atom coordinates. The purpose of this superimposition is to minimize rigid-body differences and isolate internal conformational changes.

6. The initial to final displacement vector (DiffE) is calculated. DiffE represents the experimentally observed conformational transition between structures and serves as the target vector for maximizing overlap.

7. Users select one of the following execution branches depending on the available input files. Use the structure-based mode when only initial and final structures are available or when a preliminary assessment of the conformational transition is needed. Use the trajectory-based mode when an equilibrated MD trajectory of the initial state is available, especially for flexible proteins or when conformational fluctuations are expected to improve the response prediction.

a. Structure-based mode (if no DCD file is provided):

i. A coarse-grained elastic network model (ENM) is constructed using ProDy for protein representation.

ii. The Hessian matrix (**H**) is calculated with a cutoff distance of 12 Å.

iii. The inverse of the Hessian matrix is computed for further analysis.

**Caution:** The cutoff distance should not be modified unless there is a physics-based reason for the problem at hand; we recommend it based on prior convergence analyses [31].

b. Trajectory-based mode (if a DCD file is provided):

i. Use the molecular dynamics (MD) trajectory corresponding to the initial structure.

ii. Use the equilibrated portion of the MD trajectory (e.g., 40 ns), determined by RMSD plateau analysis (see [32] for details).

iii. Load the MD trajectory for the initial structure and select C_α_ atoms.

iv. The initial.dcd file must have the same atom ordering as initial.pdb.

v. Compute the mean structure and the deviation for each frame from the trajectory.

vi. Using these deviations, the covariance matrix (**C**) is constructed.

**Caution:** It is essential to use only equilibrated trajectory segments to avoid bias from transient relaxation effects. These effects can bias overlap calculations.

8. All matrices and displacement vectors are stored automatically for downstream analysis.

**Pause point:** The protocol can be paused after this section. All required inputs for MPR overlap calculations are saved and can be reused without repeating previous steps.

**Output:**

initial_hessian.dat: Hessian matrix constructed in the structure-based mode.

initial_inv_hessian.dat: Contains the inverse Hessian matrix in the structure-based mode; in the trajectory-based mode, it stores the covariance matrix computed from MD simulations.

diffE.dat: Initial–final displacement vector constructed based on an elastic network; stored as a numerical array with dimension 3*N* × 1, where *N* is the number of C_α_ atoms used in the calculations.

**C. Overlap calculation**

*Note: This section identifies the perturbed residues that best reproduce the reference conformational transition and, by extending the analysis to multiple perturbations, provides residue combinations and the force-vector dimension required for the transition.*

1. The pipeline uses the displacement definition depending on the selected execution branch used in section B.

2. The minimum and maximum number of perturbed residues (*k*_min_ and *k*_max_) must be specified; default runs use *k* = 1–5. Start with *k* = 1 to obtain the single-residue solution and then increase *k* to evaluate whether multiple residues improve the overlap. In practice, *k* should be increased until the overlap values reach a plateau or until the improvement becomes marginal.

3. Execute the notebook cell:

a. The code computes the overlap between experimental and predicted displacement vectors.

b. There are two overlap calculation methods: enumeration-based and optimization-based.

c. Enumeration-based analysis is applied for *k* = 1–2.

d. Optimization-based MPR is used for *k* ≥ 3.

e. Results from *k* = 1–2 are first evaluated, and average force magnitudes and overlap values obtained from these enumeration runs are used to estimate the optimization scaling parameter *M*. These enumeration runs provide a reasonable range for force magnitudes and help reduce numerical instability during optimization.

**Caution:** The optimization-based method uses a mixed-integer quadratic programming formulation and requires a Gurobi Optimizer license.

4. The maximum overlap value (*O*_max_) is computed by comparing predicted displacements with the experimental displacement vector.

a. Higher *O*_max_ values indicate better reproduction of the observed conformational transition.

b. When low *O*_max_ values are observed for *k* = 1, this indicates that cooperative multi-residue perturbations are required to capture the transition.

**Output:**

results.txt: This text file contains all MPR outputs, including residue indices, force vectors, and overlap values for each *k*.

**D. Residue determination and force vector processing**

*Note: This section converts numerical MPR outputs into spatially interpretable force representations. To achieve this, the coordinates of the initial structure are combined with the force vectors obtained from the MPR analysis to calculate the positions and directions of the vectors that will be displayed on the protein structure. The resulting force representations are prepared for use in subsequent structural visualization steps.*

1. The residue indices and *O*_max_ values in results.txt are compared for all *k* values to identify the maximizing residues.

2. To visualize the MPR results for the residue indices associated with the highest overlap values, run the vector-processing section within the notebook.

3. The optimized force vector corresponding to the specified residue index and overlap value is retrieved from the output file in vector form.

4. Using the specified residue indices, the corresponding residue coordinates are extracted from the initial.pdb file.

5. The final coordinates are computed by adding the force vectors to the initial coordinates.

6. Force vectors for the best solutions (10 by default) for each *k* are represented as arrows to visualize the direction and magnitude of perturbations.

7. All .bild files are stored in a dedicated output folder.

**Output:**

.bild: Compatible with ChimeraX.

**E. Structural visualization**

*Note: The qualitative evaluation of MPR results is provided through three-dimensional visualization.*

1. Launch ChimeraX and load the initial and final structures.

2. Using the Morph command, the conformational transitions are visualized from the initial to the final structure.

3. The .bild file is loaded directly into ChimeraX using the open command, which automatically displays the force vectors as arrows and the maximizing residues as spheres.

4. Results can be exported as a static image or as morph movies.

**Result interpretation**

The overlap metric (*O*_max_) is an important parameter for evaluating the results. This metric measures how well the displacement vector predicted by the applied perturbations reproduces the conformational change between the initial and final structures. It is the normalized inner product between the predicted response vector and DiffE, ranging from 0 (mismatch) to 1 (perfect fit). The maximum overlap among all tested perturbation combinations is reported as *O*_max_. This value is calculated for each value of *k* (the number of perturbed residues). Higher *O*_max_ values indicate that the structure obtained with the chosen perturbation direction and magnitude better matches the target final structure. The interpretation of *O*_max_ should be based on trends across *k* values. A higher value for *k* = 1 suggests that a single dominant perturbation can reproduce the transition, whereas an increase at higher *k* values indicates cooperative multi-residue control. If *O*_max_ remains low for all tested *k* values, structural alignment, residue matching, trajectory equilibration, and the physical relevance of the selected structures should be checked. As a general rule, we seek *O*_max_ values exceeding 0.65 to claim the allosteric nature of the transitions that may be induced by perturbing the associated residues.

The parameter *k* controls how many residues are perturbed simultaneously in the MPR calculation. Single-residue perturbations (*k* = 1) correspond to PRS and are suitable for localized allosteric effects dominated by a single hotspot. For proteins undergoing moderate domain motions or hinge-like changes, convergence of *O*_max_ values is achieved at lower *k* values (2–3). However, for large-scale or highly cooperative conformational transitions—such as domain opening/closing or inter-domain reorientation—*k* ≥ 4 should generally be considered. During model implementation, *k* should be increased incrementally until *O*_max_ reaches a plateau. In practice, *O*_max_ values above 0.6–0.7 typically indicate a reasonable representation of the transition, but relative comparisons between different *k* values are more informative than absolute thresholds.

The residue indices reported in the MPR output start at 1 and increase sequentially along the protein chain. Although the automatically generated visualization files account for this indexing, it is important to note that when examining the results file, the numbering may not match the residue numbers in the PDB if the PDB does not start at residue 1. Because the starting residue in the PDB file corresponds to residue 1 in the MPR results, the actual residue numbers corresponding to the residues that yield the maximum overlap can be obtained by applying the appropriate offset. Finally, it should also be kept in mind that the results point out approximate regions that would trigger the conformational change being represented, rather than exact residue positions. This is because of the coarse graining involved and the linear response approximation used in the calculations. Therefore, residues that are spatially close to those pointed out by MPR might also be informative on the physics underlying the transition.

## Validation of protocol

We selected the validation examples to represent distinct practical use cases of the MPR protocol rather than to provide an exhaustive benchmark across protein families. Calmodulin (CaM, a calcium-binding protein) was chosen as a well-characterized protein with large conformational diversity and cooperative domain/lobe motions. OXA-23 (a carbapenem-hydrolyzing β-lactamase) was chosen to illustrate an environmentally driven conformational transition, in this case associated with pH-dependent structural changes. The previously published biosensor-design applications were included to demonstrate that MPR-selected residues can correspond to experimentally used functional sites in sensor engineering. Together, these examples test the protocol across different types of conformational transitions and input scenarios.

The protocol was evaluated at three levels: consistency across *k* values, comparison between structure-based and trajectory-based modes, and biological relevance. Consistency was assessed by comparing overlap values as *k* increased. Structure-based and trajectory-based analyses were compared when MD trajectories were available. Biological relevance was evaluated by mapping the identified residues onto known functional or structurally characterized regions.

For this purpose, we tested proteins that undergo various conformational transitions. The MPR method identifies combinations of amino acids that maximize structural overlap. This approach utilizes either structural differences between initial and final states or conformational fluctuations derived from molecular dynamics simulations. As representative systems, we analyzed calmodulin and OXA-23.

To begin with, the method was tested on CaM to demonstrate its effectiveness even in the presence of broad conformational diversity. This small protein, containing 148 amino acids, has a central linker connecting two globular lobes. Each unit contains a pair of homologous domains (HDs) that bind calcium [33]. It mediates many important cellular processes, including apoptosis (programmed cell death), neuronal signaling, cell proliferation, and muscle contractions, and binds many proteins during these cellular processes [34]. Due to these diverse binding functions, it can adopt different conformations through interactions with other proteins and calcium ions (Ca^2+^). Possible conformations include the *apo* form (where calcium is not bound, the lobes are closed, and the hydrophobic target binding pockets are hidden), partially calcium-bound intermediate states (only N or C-terminal bound, one lobe is open and the other is half-closed), and the calcium-bound *holo* form (both lobes are open, hydrophobic surfaces are exposed for binding to target proteins) [35]. Because of this variety, a rich database of structural (atomic coordinates of protein models) and experimental data (such as binding, kinetic, or mutational analyses) is available to validate the model.

Moreover, high sequence similarity between initial and final structures is required for effective use of the MPR method. We screened calmodulin structures across conformations to meet this requirement and selected 3CLN (open) and 1PRW (closed). First, we attempted a structure-based MPR approach using the initial and final structures. Under these conditions, the overlap values we obtained were limited. To achieve better values, we applied a trajectory-based model that was not solely based on static structure. To this end, we performed molecular dynamics simulations of these structures and focused on the plateau regions identified from RMSD analyses. We measured overlaps with the experimental *apo–holo* displacement vector and found that overlap values increased substantially under multiple perturbations. A comparison of structure-based and trajectory-based models on CaM is given in Table 1.

**Table 1.** Comparison of overlap values (*O*_max_) from structure-based and trajectory-based models for calmodulin

Model type	*k* = 1	*k* = 2	*k* = 3
Structure-based (static ENM)	0.495	0.555	0.592
Trajectory-based (MD covariance)	0.559	0.585	0.599

**Figure 1.**
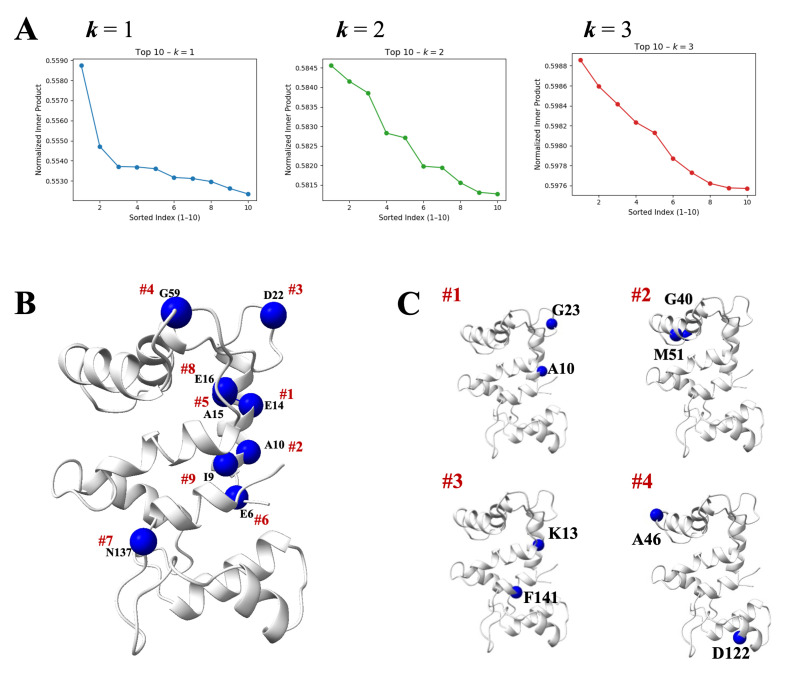
Calmodulin Multiply Perturbed Response (MPR) results. (A) The top 10 solutions ranked by overlap for *k* = 1, *k* = 2, and *k* = 3. (B) The positions of the top 10 residues for *k* = 1 solutions (force-vector directions not shown). (C) The four best *k* = 2 solutions mapped onto the protein.

Figure 1 displays the outcomes we achieved with the trajectory-based approach. The *k* = 1, *k* = 2, and *k* = 3 overlap values are shown in Figure 1A. This increase suggests that the transition is governed by collective mechanisms involving multiple residues. Figure 1B–C highlights the identified residues, which cluster in the N-terminal lobe. Previous studies show that the N-lobe plays a dominant role in CaM dynamics [36]. Consistent with this, residues identified by MPR are spatially clustered, indicating that the method produces physically meaningful allosteric communication pathways.

**Figure 2.**
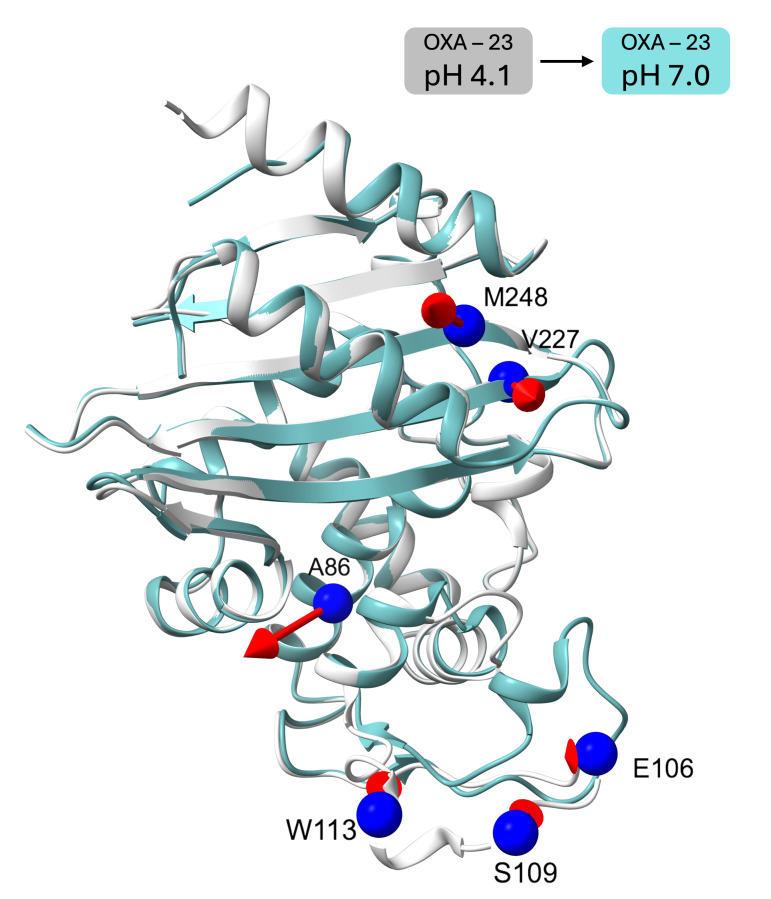
OXA-23 Multiply Perturbed Response (MPR) results. The transition from the acidic form (pH 4.1) to the neutral form (pH 7.0) was analyzed using the MPR framework. The six best single-residue perturbation solutions (*k* = 1), ranked by overlap with the experimental displacement vector, are visualized on the protein structure. Blue spheres indicate residues identified as overlap maximizers, while arrows represent the corresponding MPR-derived force vectors applied at these sites.

The second protein used for validation is the pH-dependent enzyme OXA-23. We sought to demonstrate the method's ability to capture environmentally dependent conformational transitions by investigating conformational shifts driven by changes in protonation state, independent of ligand binding. OXA-23 is the most frequently encountered OXA enzyme in isolates of *Acinetobacter baumannii*, a common hospital-acquired pathogen known for its high antimicrobial resistance and ability to form biofilms that enable it to withstand challenging environments [37,38]. *A. baumannii* is classified as one of the ESKAPE organisms, a group of bacteria responsible for a substantial proportion of resistant infections in clinical medicine that require multidisciplinary treatment strategies [39]. The production of the OXA-23 enzyme is key in the development of bacterial resistance to penicillin, cephalosporins, and carbapenems. In cases of excessive enzyme production, a significant increase in bacterial resistance to antibiotics such as ampicillin and imipenem has been observed [40]. Therefore, understanding the mechanism of action of this enzyme is important. We examined experimentally determined OXA-23 structures at different pH values. In our analysis, the structure obtained at pH 4.1 (4JF5) was used as the initial state, and that at pH 7.0 (4JF6) as the final state. After superimposing the structures, we calculated the displacement vector and evaluated overlap values for different perturbation magnitudes. As shown in Figure 2, overlap-maximizing residues were identified for the pH 4.1-to-pH 7.0 transition. For a single perturbation (*k* = 1), the maximum overlap was approximately 0.75, indicating that the pH-dependent conformational change is already well-captured. For *k* = 2, the maximum overlap increased to 0.80, with residue 113 and neighboring residues repeatedly appearing among the solutions. For triple perturbations (*k* = 3), the overlap further increased to *O*_max_ ≈ 0.82, revealing spatially close residues such as 106, 109, and 113. Since the same residues were found even when *k* was increased, only the results for *k* = 1 are shown in Figure 2. It is known that the active site of OXA-23 is accessed via the hydrophobic bridge residues Phe110 and Met221 [41]. This bridge narrows the entrance to the active site, creating a barrier that allows only specific substrates to access it. When a substrate binds, a channel opens due to motions in this hydrophobic region. A molecule that stabilizes the structure or dynamics of this region can prevent substrate entry by creating an allosteric effect. Consistent with this, our results include residues near the hydrophobic bridge, and other residues identified by MPR have been reported as active-site residues in the literature. For example, Arg259, which we obtained in our results, is reported as an essential active-site residue in OXA-23 [42]. We also detect Ala86 in our *k* = 2 analyses. It is spatially close to the conserved Ser79–Thr80–Phe81–Lys82 motif, especially the carboxylated Lys82, which is required for serine acylation as a general base and is located in the center of the active site [43]. The detection of these residues shows that our method yields results that are consistent with the enzyme's catalytic core and the dynamic regions controlling substrate entry. Overall, these findings demonstrate that the MPR protocol can reliably capture not only ligand-dependent transitions but also conformational changes triggered by environmental factors such as pH.

This protocol has also been validated using data from our previously published study on fluorescent biosensor design using the MPR approach [19]. In that study, overlap-maximizing residues determined by MPR for single fluorescent protein-based sensors coincide with the experimentally used fluorescent-protein splice sites in CaM–M13 (NCaMP7) and citrate-binding protein (CitAP, CitrON) sensors. Furthermore, the applicability of the protocol to FRET-based sensor architectures is demonstrated therein using the mlCNBD sensing domain. Overall, the MPR protocol can reliably identify functional allosteric regions in different biosensor design strategies.

These examples support the practical applicability of MPR, but they should not be interpreted as a comprehensive benchmark of all possible protein classes or transition types. The performance of MPR may depend on the quality of the input structures or trajectories, the magnitude and nature of the conformational change, residue matching between states, and the suitability of the linear-response approximation for the system under study. Therefore, for new systems, users should interpret MPR predictions as candidate allosteric regions and, where possible, compare them with independent structural, biochemical, mutational, or simulation-based evidence.

This protocol or parts of it has been used and validated in the following research article(s):

• Berksoz et al. [19]. Multiply Perturbed Response to Disclose Allosteric Control of Conformational Change: Application to Fluorescent Biosensor Design. *J Mol Biol.* 437(20): 169234. https://doi.org/10.1016/j.jmb.2025.169234

## General notes and troubleshooting

**General notes**

1. Residue indexing starts at 1 and extends to the final residue, as this is a feature of elastic network models.

2. Initial and final structures must contain the same number of C_α_ atoms for reliable displacement vector calculations.

3. When AlphaFold models are used, reduced MSA depth may be required to obtain *apo*-like conformations, especially for highly interacting proteins.

4. MD trajectories should be selected from the time interval when the system reaches equilibrium. Plateau regions (typically 40–50 ns) should be identified using RMSD analyses, and the covariance matrix should be calculated from these regions.

5. Trajectories containing enough time frames (typically larger than 3*N*) should be used to ensure reliable calculation of the covariance matrix. Trajectories that are too short or insufficiently sampled can lead to noisy covariance matrices and low overlap values.

6. The *apo* and *holo* structures used in structure-based mode should reflect the same experimental conditions or comparable computational protocols as much as possible. Large gaps between structures, differences in chain length, or differences in resolution can complicate the interpretation of displacement vectors.

7. Interpretation of maximizer residues should consider that the model is coarse-grained, and identified residue indices indicate approximate regions.

**Troubleshooting**

**Problem 1:** Low *O*_max_ values for single-residue perturbations.

Possible cause: The conformational change requires multi-site perturbations.

Solution: Apply MPR with *k* ≥ 2 using the enumeration or optimization approach.

**Problem 2:** Noisy covariance matrix or low/unstable *O*_max_ values in trajectory-based mode.

Possible causes: The MD trajectory may be too short, insufficiently sampled, or may include unequilibrated frames. A small number of frames relative to the number of degrees of freedom can produce a poorly estimated covariance matrix.

Solutions: Use only the equilibrated portion of the trajectory, as judged from RMSD or other stability metrics. Increase the number of frames when possible and avoid using early relaxation periods. As a practical check, trajectory segments should contain enough frames to estimate fluctuations reliably, and the resulting *O*_max_ values should be compared across independent or non-overlapping trajectory windows when available.

**Problem 3:** Alignment problems or unexpectedly low *O*_max_ values after structure preparation.

Possible causes: Initial and final structures may contain different residue ranges, missing loops, chain mismatches, or inconsistent C_α_ atom ordering.

Solutions: Align the sequences, retain only the common residue range, remove unresolved or nonmatching residues, and verify that the C_α_ atoms are in the same order in both structures.

**Problem 4:** The optimization approach fails to converge or gives unstable solutions.

Possible causes: The scaling parameter *M* may not be correctly estimated, *k* may be too large, or the solver settings may be too restrictive.

Solutions: Estimate *M* using enumeration runs from *k* = 1 to *k* = 2 before performing optimization. If the issue persists, reduce *k*_max_, adjust the solver time limit, and compare the returned overlap values and residue combinations.

**Problem 5:** For larger values of *k*, the optimization approach requires more computational time.

Possible cause: As *k* increases, the combinatorial search space grows rapidly, and the mixed-integer programming (MIP) may take a long time to prove optimality.

Solution: Set a time limit for the solver so it terminates after a reasonable runtime and returns the best solution found so far, even if optimality has not been proven. The solver will also report an optimality gap, indicating how close the returned solution is to the true optimum.
